# Cost-Related Prescription Drug Rationing by Adults With Obesity

**DOI:** 10.1001/jamanetworkopen.2024.33000

**Published:** 2024-11-05

**Authors:** Alissa S. Chen, Caroline G. Borden, Maureen E. Canavan, Joseph S. Ross, Carol R. Oladele, Kasia J. Lipska

**Affiliations:** 1 National Clinician Scholars Program, Department of Internal Medicine, Yale School of Medicine, New Haven, Connecticut; 2 Yale School of Medicine, New Haven, Connecticut; 3 Yale Cancer Outcomes, Public Policy, and Effectiveness Research Center, Yale School of Medicine, New Haven, Connecticut; 4 Section of General Internal Medicine, Department of Internal Medicine, Yale School of Medicine, New Haven, Connecticut; 5 Department of Health Policy and Management, Yale School of Public Health, New Haven, Connecticut; 6 Center for Outcomes Research and Evaluation, Yale New Haven Health System, New Haven, Connecticut; 7 Equity Research and Innovation Center, Yale School of Medicine, New Haven, Connecticut; 8 Section of Endocrinology, Department of Internal Medicine, Yale School of Medicine, New Haven, Connecticut

## Abstract

This cross-sectional study analyzes data from the National Health Interview Survey to examine the prevalence of cost-related prescription drug rationing by adults with obesity.

## Introduction

The out-of-pocket costs associated with treating obesity and secondary conditions, such as cardiovascular disease (CVD), can be prohibitive.^1^ When patients cannot afford these treatments, they often resort to cost-related prescription drug rationing.^2^ Glucagon-like peptide-1 receptor agonists (GLP-1RAs) offer benefits in treating both obesity and CVD,^3^ but given their high prices,^4^ patients struggling with cost-related prescription rationing will be unable to access new therapies. Understanding prevalence of cost-related prescription drug rationing among people with obesity can inform expectations about adopting these medications.

## Methods

We analyzed 2020 to 2022 data from the National Health Interview Survey, a nationally representative cross-sectional survey of US households (eMethods in Supplement 1). We included adults who were not pregnant, did not have diabetes, used at least 1 prescription medication, and answered all 3 prescription drug rationing questions. Cost-related prescription rationing was defined as any self-reported skipping, taking less, or delaying filling a medication to save money. Prevalence of rationing was calculated in people with and without obesity and within subgroups defined by age, sex, race and ethnicity (self-reported), income, health insurance coverage, CVD, and cancer for adults with obesity. Because obesity and cost-related drug rationing vary by race and ethnicity, this variable was analyzed (eMethods and eTable 1 in Supplement 1). The Yale University Institutional Review Board approved this cross-sectional study and waived informed consent because it was not considered human participant research. We followed the CROSS reporting guideline.

Statistical comparisons were made using χ^2^ tests. We calculated estimated probabilities from a multivariable logistic regression model of cost-related prescription rationing for people with obesity within subgroups defined by sociodemographic and clinical characteristics (eTable 2 in Supplement 1). All analyses used survey weights to account for the complex survey design used to ascertain nationally representative estimates. Analysis was performed using Stata, version 18 (StataCorp LLC). Two-sided *P* < .05 was considered significant.

## Results

The analytical sample comprised 51 720 adults (mean [SD] age, 51.2 [19.1] years; 30 230 females [58.4%]; 5040 Hispanic [9.7%]; 46 680 other ethnicities [90.3%]; 323 American Indian or Alaska Native [0.6%]; 2288 Asian [4.4%]; 5015 Black [9.7%]; 41 380 White [80.0%]; 1041 individuals [2.0%] of other races; and 1673 with missing data [3.2%]), representing 138 603 574 US adults. Of those, 16 768 adults (mean [SD] age, 50.1 [16.6 years]; 55.8% female; 1677 [10.0%] with self-reported CVD) or 33.9% (95% CI, 33.2%-34.5%) had obesity, representing 46 921 106 US adults.

Prevalence of cost-related prescription drug rationing among adults with vs without obesity was 8.3% (95% CI, 7.7%-8.8%) vs 5.9% (95% CI, 5.6%-6.2%; *P* < .001). Individuals with obesity and CVD had higher prevalence of rationing than those without CVD (10.3% [95% CI, 8.8%-12.0%] vs 8.0% [95% CI, 7.5%-8.6%]; *P* = .005). Prevalence of rationing was also higher among patients with obesity who were younger, female, had lower income levels, and were uninsured (Table 1). In fully adjusted analyses, age 18 to 44 years, female sex, lower income, lack of health insurance coverage, and CVD were associated with higher estimated probability of rationing among individuals with obesity (Table 2). The adjusted estimated probability of cost-related prescription drug rationing was 7.4% (95% CI, 5.5%-9.3%) for those with CVD and 4.4% (95% CI, 3.6%-5.3%) for those without CVD.

**Table 1.** Prevalence of Self-Reported Cost-Related Prescription Drug Rationing by Adults With Obesity

Characteristic	Participants		Prevalence,% (95% CI)	*P* value
	Unweighted, No. (%)	Weighted, No.^a^		
Self-reported cost-related prescription drug rationing	1313	3 872 238	8.3 (7.7-8.8)	NA
Age, y				
18-44	542 (41.3)	1 875 958	10.3 (9.3-11.3)	<.001
45-64	551 (42.0)	1 555 696	8.5 (7.7-9.3)	
≥ 65	217 (16.5)	435 439	4.3 (3.6-5.0)	
Missing data	3 (0.2)	5146	14.6 (19.0-43.2)	
Sex				
Male	400 (30.5)	1 236 657	6.0 (5.2-6.8)	<.001
Female	913 (69.5)	2 635 581	10.0 (9.3-10.9)	
Race				
American Indian or Alaska Native	15 (1.1)	33 452	7.8 (3.4-14.8)	<.001
Asian	12 (0.9)	30 508	4.4 (1.7-8.9)	
Black	230 (17.5)	678 210	9.8 (8.5-11.3)	
White	957 (72.9)	2 727 945	7.7 (7.1-8.4)	
Other^b^	33 (2.5)	114 754	11.2 (7.2-16.3)	
Missing data	66 (5.0)	287 369	11.6 (8.6-15.2)	
Ethnicity				
Hispanic	200 (15.2)	713 781	10.7 (9.0-12.5)	<.001
Other ethnicities^c^	1113 (84.8)	3 158 456	7.9 (7.3-8.4)	
Federal poverty level				
0 to <100%	224 (17.1)	623 719	13.3 (11.2-15.7)	<.001
100% to <200%	366 (27.9)	1 085 432	13.0 (11.4-14.7)	
200% to <400%	481 (36.6)	1 495 968	10.1 (9.0-11.3)	
≥400%	242 (18.4)	667 118	3.5 (*3.0-4.1)	
Health insurance coverage				
Private	687 (52.3)	2 020 656	6.9 (6.3-7.5)	<.001
Medicare	123 (9.4)	241 764	5.2 (4.2-6.4)	
Medicaid	214 (16.3)	611 749	8.9 (7.6-10.4)	
Uninsured	207 (15.8)	776 829	26.3 (22.1-30.9)	
Other^d^	82 (6.2)	221 240	7.4 (5.7-9.4)	
Cardiovascular disease	191 (14.5)	481 690	10.3 (8.8-12.0)	.005
Cancer	153 (11.7)	386 026	7.4 (6.1-8.9)	.22

Abbreviation: NA, not applicable.

^a^ Weighted numbers were rounded; therefore, numbers in some of the categories may not sum to 3 872 238.

^b^ Other included multiracial and other single races, which were not further defined by the data source.

^c^ Other ethnicities included in this category were not defined by the data source.

^d^ Other health insurance coverages included in this category were not defined by the data source.

**Table 2.** Adjusted Estimated Probability of Self-Reported, Cost-Related Prescription Drug Rationing by Adults With Obesity

Characteristic	Estimated probability, % (95% CI)
Age, y	
18-44	4.8 (3.9-5.7)
45-64	4.4 (3.6-5.3)
≥ 65	1.7 (1.1-2.2)
Missing	7.9 (0-18.5)
Sex	
Male	2.8 (2.3-3.4)
Female	4.4 (3.6-5.3)
Race	
American Indian or Alaska Native	2.8 (0.5-5.1)
Asian	2.8 (0.6-4.9)
Black	4.2 (3.2-5.2)
White	4.4 (3.6-5.3)
Other^a^	5.1 (2.8-7.4)
Missing	4.5 (2.6-6.4)
Ethnicity	
Hispanic	4.0 (2.8-5.3)
Other ethnicities^b^	4.5 (3.7-5.3)
Federal poverty level	
0% to <100%	15.1 (11.7-18.5)
100% to <200%	14.6 (12.0-17.2)
200% to <400%	11.7 (9.9-13.6)
≥400%	4.4 (3.6-5.3)
Health insurance coverage	
Private	4.4 (3.6-5.3)
Medicare	5.5 (3.5-7.5)
Medicaid	3.1 (2.2-4.0)
Uninsured	11.9 (8.5-15.3)
Other^c^	3.9 (2.7-5.1)
Cardiovascular disease	
Absent	4.4 (3.6-5.3)
Present	7.4 (5.5-9.3)
Cancer	
Absent	4.4 (3.6-5.3)
Present	5.2 (3.8-6.7)

^a^ Other included multiracial and other single races, which were not further defined by the data source.

^b^ Other ethnicities included in this category were not defined by the data source.

^c^ Other health insurance coverages included in this category were not defined by the data source.

## Discussion

Patients with obesity, particularly those with CVD, were more likely to engage in cost-related prescription drug rationing, suggesting difficulties affording their medications. Given that few insurance providers cover GLP-1RA for obesity,^5^ cost-related prescription drug rationing could be exacerbated if patients were prescribed GLP-1RAs at their current prices (over $1000 per month).^4^ Moreover, high prices of GLP-1RAs may worsen health disparities because Black and Hispanic individuals with obesity have a higher prevalence of prescription drug rationing compared with White and non-Hispanic individuals.

Study limitations include inability to assess which medications adults were prescribed and whether adults were already prescribed GLP-1RA for treating obesity, although use for indications other than diabetes was minimal until late 2022.^6^ Respondent answers to survey questions are subject to recall bias, and weight and height were self-reported.

## Notes

Supplement 1.
eMethods.
**eTable 1.** Combined Groups for Health Insurance Coverage **eTable 2.** Mode for Each Covariate Used for Marginal Standardization Prediction
eReferences.

Supplement 2.
Data Sharing Statement
