# Supplementary material for: Cost-Related Prescription Drug Rationing by Adults With Obesity
Source: JAMA Netw Open. 2024 Nov 5;7(11):e2433000. doi: 10.1001/jamanetworkopen.2024.33000 (PMC11539006; doi:10.1001/jamanetworkopen.2024.33000)
Supplement: Supplement 1. — eMethods. eTable 1. Combined Groups for Health Insurance Coverage eTable 2. Mode for Each Covariate Used for Marginal Standardization Prediction eReferences. [file jamanetwopen-e2433000-s001.pdf]

Supplementary Online Content

Chen AS, Borden CG, Canavan ME, Ross JS, Oladele CR, Lipska KJ. Cost-related prescription drug rationing by adults with obesity. *JAMA Netw Open.* 2024;7(11):e2433000. doi:10.1001/jamanetworkopen.2024.33000

eMethods.

eTable 1. Combined Groups for Health Insurance Coverage

eTable 2. Mode for Each Covariate Used for Marginal Standardization Prediction

eReferences

This supplementary material has been provided by the authors to give readers additional information about their work.

© 2024 Chen AS et al. *JAMA Network Open*.

eMethods

Survey

The National Health Interview Survey (NHIS) is a cross-sectional survey of the United States households conducted by the National Center for Health Statistics.[1] The target population is civilian non-institutionalized U.S. population. The NHIS is conducted by trained interviewers who survey households in person or by telephone if in person is not an option. Interviewers use computers to assist their interviewing and prevent error. The questions asked are changed every year, but interviewers survey households every month of the year. The survey takes about an hour to complete. The NHIS uses a geographically clustered sampling technique to select households to participate in the survey. This means households are chosen based on their characteristics (e.g. age, sex, race) to ensure that the entire population of the United States is represented in the data. The NHIS uses complex survey design, so analyses are weighted in order to create nationally representative estimates.[2] Questions included in the NHIS are evaluated by the National Center for Health Statistics' Collaborating Center for Questionnaire Design and Evaluation Research using cognitive interviewing methods.[3] As part of this method, respondents are asked their thought process as they respond to questions, which allows researchers to understand construct validity and any issues respondents might have. Questions are re-evaluated on an ongoing basis.

Participants

Adults were included in the analytic sample if they were aged 18 years of age or older, they were not pregnant, had self-reported height and weight, did not have diabetes, used prescription drugs, and answered all three cost-related prescription drug rationing questions. Pregnancy status was ascertained from the question "are you currently pregnant?". Use of prescription drugs was ascertained from the question "at any time in the past 12 months, did you take prescription medication?" History of diabetes

© 2024 Chen AS et al. *JAMA Network Open*.

was ascertained from the question "has a doctor or other health professional ever told you that you had diabetes?" For participants who previously had gestational diabetes or prediabetes, they were asked "Not including gestational diabetes/prediabetes, has a doctor or other health professional ever told you that you had diabetes?"

Measures

Cost-related prescription drug rationing was assessed based on answers to three questions. The question block started "During the past 12 months, were any of the following true for you?" Participants could answer yes, no, or don't know to the following three clauses: "you skipped medication doses to save money," "you took less medication to save money," and "you delayed filling a prescription to save money." A yes to one of these questions was considered cost-related prescription drug rationing.

Obesity was defined as a body mass index of 30 kg/m^2 or higher calculated using self-reported height and weight.

Sex was ascertained from the question "Are you male or female?"

Health coverage status was obtained from participants using questions "Are you covered by any kind of health insurance or some other kind of health care plan?" "What kinds of health insurance or health care coverage do you have?" Participants were provided with examples of insurance (e.g. private, Medicare) if needed. Adults ≥65 additionally were asked "Are you covered by Medicare?" Adults 18-64 were categorized differently than adults aged 65 years of age and older in the public use data set. eTable 1 describes how groups were combined.

Income was ascertained from the question "What is your best estimate of total income from all sources, before taxes, in the last year?" If participants did not know their income, they were given ranges of income to choose from. Income information has historically been missing from the NHIS for a

© 2024 Chen AS et al. *JAMA Network Open*.

substantial number of patients. For example, in the 2020 interview year, income information was missing for 23% of the total sample. The NHIS provides an imputed estimate of income based on other variables in the dataset for those missing income data.[4] This analysis uses reported income and imputed income for participants without an income ascertained by the survey, as calculated by the statisticians working with the NHIS. Income was reported as percentage of the federal poverty level and categorized as 0 to <100% FPL, 100% to <200% FPL, 200% to <400% FPL, and ≥400%.

Race and ethnicity were obtained via self-report provided to the surveyors. Participants were asked "what race or races do you consider yourself to be?" Surveyors could choose White, Black, African American, American Indian, Alaska Native, Native Hawaiian, Pacific Islander, Asian, multiple races, or other race based on the participant's response. Within the multiple races and other races categories, surveyors could type in participant's responses. Other can consist of multiple races (e.g. White and Black) or other single races not in the initial list. Within the public use dataset used for this analysis, the available race categories include White only, Black/African American only, Asian only, American Indian/Alaska Native only, American Indian/Alaska Native and any other group, other single and multiple races, refused, not ascertained, don't know. The public use dataset does not state which groups are included in other single and multiple races. Groups were combined for this analysis. American Indian/Alaska Native only and any other group and other single and multiple races were combined into the other race group. Refused, not ascertained, and don't know were combined into a single missing group. For ethnicity, participants were asked "do you consider yourself to be Hispanic or Latino?" Within the public use dataset used for this analysis, the available ethnicity categories included Hispanic, non-Hispanic, refused, not ascertained, and don't know. Refused, not ascertained, and don't know were combined into a single missing group.

Race and ethnicity were included as covariates in this analysis because both obesity and cost-related prescription drug rationing is known to vary by race and ethnicity.[5,6] Importantly, race and

© 2024 Chen AS et al. *JAMA Network Open*.

ethnicity denote social constructs and are considered in this analysis along with other sociodemographic factors (e.g., income, health coverage).

Past diagnosis of cancer was added as a covariate to control for the high expense of cancer treatment.[7] Past diagnosis of cancer was ascertained from the question “Have you ever been told by a doctor or other health professional that you had cancer or a malignancy of any kind?”

Past diagnosis of cardiovascular disease was a covariate of interest in this analysis due to the high expense of cardiovascular pharmacotherapy,[8] and it is a condition that can be caused by obesity. Additionally, semaglutide was recently approved for the indication of secondary prevention of cardiovascular disease for people with obesity.[9] Past diagnosis of cardiovascular disease was defined as history of angina, myocardial infarction, stroke, and coronary heart disease. Four questions were used to ascertain these diagnoses: “Have you ever been told by a doctor or other health professional that you had angina, also called angina pectoris?”, “have you ever been told by a doctor or other health professional that you had a heart attack, also called myocardial infarction?”, “have you ever been told by a doctor or other health professional that you had a stroke?”, “have you ever been told by a doctor or other health professional that you had coronary heart disease?” An answer of “yes” to any of these questions was considered a history of cardiovascular disease.

Statistical analysis

Prevalence of cost-related prescription drug rationing was calculated for people with and without obesity. Prevalence of cost-related prescription drug rationing was also calculated within subgroups of age, sex, race, ethnicity, income, and insurance, CVD and cancer for adults with obesity. Korn-Graubard confidence intervals were calculated, which are used to create 95% confidence intervals using national survey data.[10] Using the fully adjusted logistic regression model, marginal standardization

© 2024 Chen AS et al. *JAMA Network Open*.

prediction was done at the modes of each covariate to estimate predicted probability of cost-related prescription drug rationing. The modes for each category are listed in eTable 2.

© 2024 Chen AS et al. *JAMA Network Open*.

eTable 1: Combined groups for health insurance coverage

Categorized Insurance for Ages 18-64	Categorized insurance for ages ≥65	Combined insurance category
Private	Private	Private
Medicaid and other Public	Dual eligible	Public
Other coverage	Other coverage	Other
Uninsured	Uninsured	Uninsured
Don’t know	Don’t know	Missing
	Medicare advantage	Medicare
	Medicare only excluding Medicare Advantage	Medicare

© 2024 Chen AS et al. *JAMA Network Open*.

eTable 2: Mode for each covariate used for marginal standardization prediction

Category	Mode
Age	45-64
Sex	Female
Race	White
Ethnicity	Non-Hispanic
Percent federal poverty level	≥400%
Health insurance	Private
Cardiovascular disease diagnosis	Absent
Cancer diagnosis	Absent

© 2024 Chen AS et al. *JAMA Network Open*.

eReferences

1. Centers for Disease Control and Prevention. About the National Health Interview Survey. Updated November 22, 2023. April 24, 2024. https://www.cdc.gov/nchs/nhis/about_nhis.htm

2. Centers for Disease Control and Prevention. About NHIS. Accessed June 17, 2024. https://www.cdc.gov/nchs/nhis/about_nhis.htm

3. Wilmot A, Creamer L. *Cognitive Interview Evaluation of Questions for Inclusion on the 2023 National Health Interview Survey - CCQDER*. 2023. https://wwwn.cdc.gov/qbank/report.aspx?1238

4. Centers for Disease Control and Prevention. *Multiple Imputation of Family Income in 2021 National Health Interview Survey: Methods*. Updated August 2022. https://ftp.cdc.gov/pub/Health_Statistics/NCHS/Dataset_Documentation/NHIS/2021/NHIS2021-imputation-techdoc-508.pdf

5. Centers for Disease Control and Prevention. Adult Obesity Facts. Accessed July 28, 2023, https://www.cdc.gov/obesity/php/data-research/adult-obesity-facts.html?CDC_AAref_Val=https://www.cdc.gov/obesity/data/adult.html

6. Laryssa M, Cohen RA. *Characteristics of Adults Aged 18–64 Who Did Not Take Medication as Prescribed to Reduce Costs: United States, 2021*. https://stacks.cdc.gov/view/cdc/127680

7. Yabroff KR, Lund J, Kepka D, Mariotto A. Economic burden of cancer in the United States: estimates, projections, and future research. *Cancer Epidemiol Biomarkers Prev*. Oct 2011;20(10):2006-14. doi:10.1158/1055-9965.Epi-11-0650

8. Khera R, Valero-Elizondo J, Nasir K. Financial Toxicity in Atherosclerotic Cardiovascular Disease in the United States: Current State and Future Directions. *Journal of the American Heart Association*. 2020;9(19):e017793. doi:doi:10.1161/JAHA.120.017793

9. U.S. Food and Drug Administration. FDA Approves First Treatment to Reduce Risk of Serious Heart Problems Specifically in Adults with Obesity or Overweight. Accessed June 16, 2024. https://www.fda.gov/news-events/press-announcements/fda-approves-first-treatment-reduce-risk-serious-heart-problems-specifically-adults-obesity-or

10. Ward BW. kg_nchs: A command for Korn-Graubard confidence intervals and National Center for Health Statistics' Data Presentation Standards for Proportions. *Stata J*. Sep 2019;19(3):510-522. doi:10.1177/1536867x19874221

© 2024 Chen AS et al. *JAMA Network Open*.
