# Supplementary material for: Cost-Related Prescription Drug Rationing by Adults With Obesity
Source: JAMA Netw Open. 2024 Nov 5;7(11):e2433000. doi: 10.1001/jamanetworkopen.2024.33000 (PMC11539006; doi:10.1001/jamanetworkopen.2024.33000)
Supplement: Supplement 2. — Data Sharing Statement [file jamanetwopen-e2433000-s002.pdf]

Data Sharing Statement

Chen. Cost-Related Prescription Drug Rationing by Adults with Obesity. *JAMA Netw Open*. Published November 05, 2024. doi:10.1001/jamanetworkopen.2024.33000

Data
Data available: Yes
Data types: Deidentified participant data
How to access data: Data are available from https://www.cdc.gov/nchs/nhis/index.htm
When available: With publication

Supporting Documents
Document types: None

Additional Information
Who can access the data: Anyone requesting the data
Types of analyses: Any purpose
Mechanisms of data availability: Data will be made available with investigator support.
